# A structural model of psychological vulnerability in the era of social media

**DOI:** 10.1007/s44192-026-00541-1

**Published:** 2026-07-06

**Authors:** Lisfarika Napitupulu, Ateerah Abdul Razak, Didik Widiantoro, Bahril Hidayat, Wahyu Fadilla Perkasa, Syelfi Pasha Nurfadila

**Affiliations:** 1 Faculty Psychology, Universitas Islam Riau, Jl. Kaharudin Nst, Pekanbaru, Riau 28284 Indonesia; 2 Faculty of Language Studies and Human Development, Institut Penyelidikan dan Pengurusan Kemiskinan (InspeK), Fakulti Pengajian Bahasa dan Pembangunan Insan, Universiti Malaysia Kelantan, Jalan Bendahara, Kota Bharu, Kelantan 16100 Malaysia; 3 Department of Psychology, Sultan Idris Education, Street, Tanjung Malim, Perak 35900 Malaysia

**Keywords:** Psychological vulnerability, Family functioning, Mediation

## Abstract

**Background:**

Psychological vulnerability reflects a heightened susceptibility to psychological distress when individuals encounter stressors, particularly in the absence of adequate coping resources. In the digital era, has emerged as a potential risk factor contributing to this vulnerability. Family functioning has been proposed as a protective factor; however, its moderating role in the relationship between and psychological vulnerability remains insufficiently examined.

**Methods:**

This study employed a cross-sectional quantitative design involving 284 active social media users in Pekanbaru, Indonesia, aged 18–50 years (M = 22.6, SD = 3.9), with the sample predominantly consisting of students (98.2%). Participants were recruited using a convenience sampling approach through online distribution. Data were analyzed using Partial Least Squares Structural Equation Modeling (PLS-SEM) with a disjoint two-stage approach.

**Results:**

The results indicated that was positively associated with psychological vulnerability (β = 0.400, *p* < .001), while family functioning showed a significant negative association (β = − 0.174, *p* = .001). The interaction effect between and family functioning was statistically significant but very small in magnitude (β = 0.103, *p* = .009; f² = 0.016). Conditional effect analysis showed that the association between and psychological vulnerability was stronger at higher levels of family functioning, indicating a non-buffering moderation pattern.

**Conclusions:**

These findings suggest that is an important risk factor for psychological vulnerability, whereas family functioning demonstrates a protective direct effect. However, its moderating role appears limited and does not operate in a buffering manner. Given the very small interaction effect and the use of convenience sampling with a predominantly student sample, the findings should be interpreted with caution. Future research employing longitudinal designs and more diverse samples is needed to further clarify these relationships.

**Supplementary Information:**

The online version contains supplementary material available at https://doi.org/10.1007/s44192-026-00541-1.

## Introduction

Psychological vulnerability refers to a cognitive and emotional condition characterized by increased susceptibility to psychological distress when individuals encounter stressors, particularly in the absence of adequate adaptive and coping resources [1]. From a cognitive vulnerability and stress–diathesis perspective, psychological vulnerability reflects a predispositional sensitivity that increases the likelihood of maladaptive responses when individuals encounter stressors [2]. Such maladaptive responses often manifest as difficulties in emotional regulation, heightened sensitivity to stress, and increased reliance on external validation, which collectively elevate the risk of developing mental health problems such as depression, anxiety, and post-traumatic stress symptoms [3]. Individuals with high psychological vulnerability tend to experience difficulties in emotional regulation, heightened sensitivity to stress, and increased reliance on external validation, which collectively elevate the risk of developing mental health problems such as depression, anxiety, and post-traumatic stress symptoms [4–6]. Empirical evidence consistently indicates that higher psychological vulnerability is associated with maladaptive coping strategies and poorer psychological adjustment [7–9].

The relevance of psychological vulnerability has become increasingly salient in the context of rapid digitalization and the pervasive use of social media. Social media platforms enable continuous connectivity, information exchange, and social interaction; however, excessive and uncontrolled engagement may give rise to. is commonly defined as a state of emotional exhaustion, cognitive overload, and reduced motivation resulting from prolonged exposure to online content and social demands [10–12]. Previous studies have demonstrated that and related forms of technostress are associated with diminished well-being, increased psychological strain, and emotional exhaustion across various digital platforms [13, 14]. Recent studies have further suggested that the psychological consequences of social media use may depend not only on the amount of use but also on the manner in which social media is used [15]. Active engagement, such as posting content, commenting, and interacting with others, has generally been associated with more favorable psychological outcomes and greater social connectedness [16]. In contrast, passive consumption of social media content has been linked to increased social comparison, emotional exhaustion, reduced well-being, and greater psychological distress. Emerging evidence has also indicated that temporary reductions in social media use or social media detox interventions may contribute to improvements in psychological well-being and reductions in digital stress. These findings suggest that the relationship between social media use and psychological functioning is complex and influenced by both the intensity and quality of online engagement [17].

Individuals experiencing often report attentional difficulties, emotional dysregulation, heightened anxiety, and feelings of overwhelm [18, 19, 20]. Persistent digital stressors, such as information overload and social comparison, may disrupt self-regulation and deplete psychological resources [21]. Over time, these conditions may increase individuals’ susceptibility to psychological vulnerability, positioning not only as an outcome of excessive digital engagement but also as a potential risk factor for vulnerability to psychological distress [22–24].

Nevertheless, individuals differ substantially in their psychological responses to. One factor that may explain this variability is family functioning, which refers to the quality of family relationships, communication patterns, emotional support, and problem-solving processes within the family system [25, 26]. Family functioning was selected as the focal protective factor in this study because the family represents a proximal and contextually embedded resource within individuals’ immediate social environment, serving as a primary microsystem through which individuals experience and interpret their social world [27, 28]. Unlike individual-level protective factors such as resilience or coping strategies, family functioning captures broader relational dynamics, including communication patterns, emotional support, and problem-solving processes, which collectively shape individuals’ responses to stress [29]. From a family systems and stress-buffering perspective, effective family functioning may serve as a protective resource by supporting emotional regulation and adaptive coping, thereby reducing vulnerability to digital stressors [5, 25]. Although family functioning is generally conceptualized as a protective factor, family systems theory suggests that the benefits of family cohesion are not always linear. While moderate levels of cohesion may promote support, resilience, and adaptive coping, excessively cohesive family systems characterized by diffuse interpersonal boundaries and excessive emotional involvement (i.e., enmeshment) may contribute to maladaptive outcomes and heightened stress sensitivity [30, 29]. In such contexts, emotional experiences and stress reactions may become highly interconnected among family members, potentially increasing rather than reducing vulnerability to stress. Consequently, although family functioning is typically expected to buffer the effects of stressors, family systems theory also allows for the possibility that certain forms of family involvement may amplify stress responses under specific circumstances. This perspective provides a theoretical basis for considering alternative moderation patterns beyond the traditional stress-buffering model and may help explain why higher levels of family functioning do not always correspond to stronger protective effects. In addition to its direct protective role, family functioning may also influence how individuals respond to stressors. From a stress-buffering and family systems perspective, supportive family environments can provide emotional regulation, social support, and coping resources that reduce individuals’ sensitivity to adverse experiences. In the context of, individuals with higher family functioning may be better equipped to manage cognitive overload and emotional strain, thereby attenuating the impact of digital stress on psychological vulnerability. This suggests that family functioning may not only act as a direct predictor but also as a moderating factor in the relationship between and psychological vulnerability. However, despite these theoretical perspectives, empirical evidence supporting this moderating role in the context of remains limited. Existing studies have predominantly examined, psychological vulnerability, and family functioning as separate constructs, focusing primarily on their direct associations with mental health outcomes [5, 31–33]. These lines of research therefore remain insufficiently integrated. In particular, there is a lack of empirical studies that explicitly test whether family functioning moderates the relationship between and psychological vulnerability. This gap is important, as it limits our understanding of how family context may shape individuals’ susceptibility to digital stress.

To address this gap, the present study examines a moderation model in which is hypothesized to predict psychological vulnerability, with family functioning tested as a moderating variable using a structural equation modeling approach. By clarifying these relationships, this study aims to contribute to a more nuanced understanding of individual vulnerability in the context of increasing digital stress.

Based on the theoretical framework and previous empirical findings, this study proposes a moderation model examining the relationship between, family functioning, and psychological vulnerability.

- H1: is positively associated with psychological vulnerability.
- H2: Family functioning is negatively associated with psychological vulnerability.
- H3: Family functioning moderates the relationship between and psychological vulnerability, such that higher levels of family functioning weaken the positive association between and psychological vulnerability.

## Methods

### Participants

The target population of this study consisted of individuals aged 18–50 years who were active social media users residing in Pekanbaru, Indonesia. A total of 284 participants were included in the final analysis. Participants were recruited using a non-probability sampling approach, specifically convenience sampling, through online distribution.

The sample was highly skewed, with the majority of participants being young adults (M = 22.6, SD = 3.9), predominantly students (98.2%) and female (77.1%). This demographic imbalance limits the external validity of the findings, as the results may not be generalizable to broader populations beyond young, student-dominated samples. Therefore, the findings should be interpreted with caution.

### Procedure

This study employed a cross-sectional quantitative design using an online survey. Data were collected over a four-week period through a self-administered questionnaire distributed via social media platforms, primarily WhatsApp groups and university-based online communities in Pekanbaru. Recruitment followed a convenience-based outreach strategy, in which the survey link was disseminated through student networks and personal contacts. Participation was entirely voluntary, and no incentives were provided.

Participants voluntarily completed the questionnaire after being informed about the study’s objectives, confidentiality procedures, and their right to withdraw at any time. Informed consent was obtained prior to participation, and the study was conducted in accordance with institutional ethical standards for research involving human participants.

A total of 312 responses were initially received. To ensure data quality and eligibility, a screening process was conducted by excluding incomplete responses, duplicate submissions, and participants who did not meet the age or location criteria. Following this procedure, 284 valid responses were retained for the final analysis. Data analysis was conducted using Partial Least Squares Structural Equation Modeling (PLS-SEM) with SmartPLS version 4. This approach enabled the simultaneous assessment of the measurement and structural models, including moderation analysis to examine whether family functioning influenced the relationship between and psychological vulnerability.

### Ethical considerations and online consent

Prior to accessing the questionnaire, participants were presented with an online informed consent form that clearly explained the purpose of the study, the voluntary nature of participation, confidentiality and anonymity of responses, as well as their right to withdraw at any time without penalty. Participants were required to indicate their agreement by selecting the *agree* option before proceeding to the survey, and only those who provided consent were allowed to continue. The study was conducted in accordance with institutional ethical standards for research involving human participants and received approval from the Ethics Committee of the Faculty of Psychology, Universitas Islam Riau.

### Common method bias

Given that all data were collected using self-report measures within a single survey, the potential for common method bias was considered. To assess this issue, Harman’s single-factor test was conducted. The results indicated that the first factor accounted for 21.54% of the total variance, which is below the recommended threshold of 50%. This suggests that common method bias is unlikely to pose a substantial threat to the validity of the findings. In addition to this statistical test, several procedural remedies were implemented during data collection, including assuring participant anonymity, minimizing evaluation apprehension, and separating the measurement of constructs across different sections of the questionnaire.

### Instruments

#### Cultural and linguistic adaptation of instruments

All instruments used in this study, including the Scale (SMFS), Psychological Vulnerability Scale (PVS), and Family Functioning Questionnaire (FFQ), were administered in Bahasa Indonesia. The instruments were translated from English into Bahasa Indonesia by the research team. To evaluate semantic equivalence between the translated and original versions, an AI-assisted back-translation procedure was conducted. The back-translated English versions were systematically compared with the original instruments to identify discrepancies in wording, meaning, and conceptual content. Any inconsistencies were reviewed and resolved through discussion and consensus among the research team. Through this iterative comparison and review process, efforts were made to preserve the semantic meaning of the original items while ensuring linguistic appropriateness in the Indonesian context. This procedure was intended to support semantic equivalence across language versions, which is considered an important component of cross-cultural instrument adaptation [34, 35]. Recent methodological developments suggest that AI-assisted translation and back-translation procedures may provide a feasible approach for supporting semantic consistency in questionnaire adaptation when combined with researcher review [36, 37].

The Scale (SMFS) developed by [38] was used to measure the independent variable. It consists of three dimensions—Cognitive Fatigue, Emotional Fatigue, and Behavioral Fatigue—which reflect the mental, emotional, and behavioral impacts of prolonged social media use. The 15-item scale demonstrated satisfactory internal consistency, with McDonald’s Omega (ω) = 0.83 for the overall scale and subscale reliabilities ranging from 0.61 to 0.76. Although one subscale showed reliability values close to 0.60, such levels can be considered acceptable in exploratory or early-stage applications of multidimensional scales, particularly when supported by strong overall reliability and CFA evidence. Confirmatory Factor Analysis (CFA) indicated a stable three-factor structure with good model fit indices (CFI = 0.97, TLI = 0.96, RMSEA = 0.06). Correlations among the three dimensions (*r* = .46–0.50) supported the distinct yet interrelated nature of the factors, indicating adequate convergent and discriminant validity.

#### Psychological vulnerability

Psychological vulnerability was measured using the Psychological Vulnerability Scale (PVS) developed by [39]. This 6-item instrument is rated on a 5-point Likert scale ranging from 1 (Does not describe me at all) to 5 (Describes me very well). The PVS assesses maladaptive cognitive belief patterns that increase susceptibility to stress, such as dependency on external approval, perfectionistic standards, and low self-worth. The scale demonstrated acceptable internal consistency reliability (Cronbach’s α = 0.71–0.86) across three samples and good temporal stability (test–retest *r* = .83 over 5–6 weeks). Construct validity was supported by significant positive correlations with helplessness, negative affect, and maladaptive coping behaviors, and negative correlations with optimism, self-efficacy, social support, and life satisfaction.

#### Family functioning

Family functioning was assessed using the Family Functioning Questionnaire (FFQ) developed and preliminarily validated by Roncone et al. (2007). The instrument was designed according to the cognitive-behavioral psychoeducational model and comprises three dimensions—Problem Solving, Communication Skills, and Personal Goals—which represent essential competencies targeted in family psychoeducational interventions. The final version consists of 24 items rated on a 4-point Likert scale (0 = *never* to 3 = *always*). The scale demonstrated good psychometric properties, with internal consistency reliability (Cronbach’s α = 0.84 for the total scale; α = 0.83 for Problem Solving, α = 0.71 for Communication Skills, and α = 0.66 for Personal Goals). Test–retest reliability was satisfactory (intra-class correlations > 0.75 for most items). Convergent validity was supported through positive correlations with vitality and mental health dimensions of the SF-36 and negative correlations with both objective and subjective family burden measures [40].

#### Psychometric properties of the instruments in the current sample

The psychometric properties of all measurement instruments were re-examined using the current sample (*N* = 284) following the reflective measurement model evaluation procedure recommended by [38]. Reliability and validity were assessed through Cronbach’s alpha (α), rho_A, composite reliability (ρc), and average variance extracted (AVE). Discriminant validity was further evaluated using the Fornell–Larcker criterion and heterotrait–monotrait (HTMT) ratios. Table 1 presents the HTMT ratios among all study constructs.

**Table 1:** HTMT Ratios Among Study Constructs

Construct	FF	PV	SMF	FF × SMF
FF	—	0.253	0.128	0.113
PV	0.253	—	0.676	0.269
SMF	0.128	0.676	—	0.280
FF × SMF	0.113	0.269	0.280	—

As shown in Table 1, all HTMT values ranged from 0.113 to 0.676 and were substantially below the recommended threshold of 0.85 (Henseler et al., 2015). These findings provide strong evidence of discriminant validity and indicate that Family Functioning, Psychological Vulnerability,, and the interaction construct represent empirically distinct constructs within the proposed model.

For the Scale (SMFS) which consists of 15 items across three dimensions (Cognitive, Behavioral, and Emotional Fatigue), all outer loadings exceeded the recommended threshold of 0.70, confirming indicator reliability. The scale demonstrated excellent internal consistency, with Cronbach’s α = 0.91, rho_A = 0.92, and composite reliability (ρc) = 0.94. The AVE value of 0.68 indicated adequate convergent validity. These findings confirm that the SMFS construct reliably captures the multidimensional nature of fatigue related to excessive social media use within the Indonesian young adult population.

The Family Functioning Questionnaire (FFQ) comprising 24 items representing three domains—Problem Solving, Communication Skills, and Personal Goals—also exhibited strong psychometric adequacy. All first-order dimensions achieved satisfactory reliability (α = 0.83–0.91; ρc = 0.85–0.91; AVE = 0.56–0.67). The second-order reflective construct (overall Family Functioning) showed Cronbach’s α = 0.89, rho_A = 0.90, ρc = 0.93, and AVE = 0.63, providing evidence of both internal consistency and convergent validity. These results suggest that the FFQ is a reliable and valid measure for assessing functional family dynamics in the current sample.

The Psychological Vulnerability Scale (PVS) demonstrated adequate psychometric performance, with Cronbach’s α = 0.84, rho_A = 0.85, composite reliability (ρc) = 0.88, and AVE = 0.60. Indicator loadings ranged from 0.71 to 0.88, meeting the recommended reliability criteria. The retained indicators adequately captured important aspects of psychological vulnerability, particularly maladaptive self-evaluative cognitions associated with psychological distress in social media contexts.

Although the AVE value for Psychological Vulnerability (AVE = 0.60) was lower than those observed for the other constructs, it exceeded the recommended minimum threshold of 0.50 for convergent validity [41]. In addition, the construct demonstrated satisfactory indicator loadings, composite reliability (ρc = 0.88), and internal consistency reliability, supporting its retention for subsequent structural model analysis. Nevertheless, the relatively lower AVE suggests that this construct may contain greater measurement error than the other study variables, which may slightly reduce the precision of the structural estimates.

A limitation of the Psychological Vulnerability construct should also be acknowledged. To achieve acceptable measurement properties, three indicators from the original six-item Psychological Vulnerability Scale were excluded during the measurement model evaluation process. Although the retained indicators demonstrated satisfactory reliability and convergent validity, the resulting shortened version represents a narrower operationalization of the original construct. Specifically, the retained indicators primarily reflect maladaptive self-evaluative cognitions, including negative self-worth, feelings of inferiority, and vulnerability to psychological distress under stressful conditions. These cognitive tendencies remain conceptually consistent with Sinclair and Wallston’s [39] definition of psychological vulnerability, which emphasizes vulnerability arising from maladaptive beliefs about the self. However, because the retained version no longer includes indicators reflecting dependency on external approval and perfectionistic standards, it does not fully capture the conceptual breadth of the original scale. Consequently, findings involving Psychological Vulnerability should be interpreted with appropriate caution, and future studies are encouraged to validate the complete six-item instrument in larger and more diverse Indonesian samples.

Across all instruments, the reliability coefficients (α, rho_A, and ρc) were within the recommended range of 0.70–0.95, and all AVE values exceeded 0.50, indicating satisfactory convergent validity [42]. Furthermore, the Fornell–Larcker criterion showed that the square roots of AVE for each construct were greater than their inter-construct correlations, and all HTMT ratios were below 0.85, confirming discriminant validity [43]. Collectively, these findings indicate that the measurement model demonstrated acceptable levels of reliability and validity for the reflective constructs used in this study, supporting the subsequent structural model analysis.

## Results

### Demographic characteristics

A total of 284 participants were included in this study. The sample consisted of 219 females (77.1%) and 65 males (22.9%). The age distribution indicated that the sample was predominantly composed of young adults, with the majority of participants falling within the 18–25 year age. Most participants were students (*n* = 279, 98.2%), while only 4 participants (1.4%) reported being employed. The participants’ age ranged from 18 to 50 years (M = 22.6, SD = 3.9).

The most frequently used social media platforms among participants were TikTok (33.8%) and Instagram (22.5%), followed by YouTube, Facebook, and Twitter with smaller user proportions. This reflects the predominance of short-video and visual-based platforms among young adults in Indonesia.

Although the gender composition of the sample was uneven, independent-samples t-tests indicated no significant gender differences in (t(282) = 1.42, *p* = .16) or Psychological Vulnerability (t(282) = 0.97, *p* = .33). Therefore, gender was not included as a control variable in subsequent analyses. In addition, other potential control variables such as age were examined but were not included in the structural model due to non-significant associations with the main constructs Table 2.

**Table 2:** Gender distribution of participants (*N* = 284)

Gender	*n*	%
Female	219	77.1
Male	65	22.9
Total	284	100

### Descriptive statistics and correlation analysis

Prior to testing the structural model, descriptive and correlational analyses were conducted to examine the distribution and relationships among the key study variables such as (SMF), Psychological Vulnerability (PV), and Family Functioning (FF). The results in Table 3 show that all variables demonstrated approximately normal distributions, with skewness and kurtosis values within the acceptable range (± 1).

**Table 3:** Descriptive statistics of study variables (*N* = 284)

Variable	Min	Max	Mean	SD	Skewness	Kurtosis
	18	91	56.21	13.29	–0.03	–0.02
Psychological Vulnerability	9	34	19.91	4.47	0.23	0.17
Family Functioning	26	96	65.74	15.50	–0.35	–0.25

Skewness and kurtosis values within ± 1 indicate approximate normality

Pearson’s correlation analysis, Table 4 revealed a significant positive association between and Psychological Vulnerability (*r* = .38, *p* < .001), indicating that higher levels of digital fatigue were associated with greater psychological vulnerability. Family Functioning showed weak, negative correlations with both variables, suggesting that supportive family dynamics may slightly reduce the adverse psychological effects of social media use.

**Table 4:** Pearson correlations among main study variables

Variable	1	2	3
1. Psychological Vulnerability	—		
2.	0.382***	—	
3. Family Functioning	–0.095	–0.089	—

**p < .001

#### Measurement model analysis (outer model analysis)

The scale in this study was developed by [38]to measure a model of psychological vulnerability by involving three main constructs: Family Functioning (FF), Psychological Vulnerability (PV), and (SMF). The Family Functioning (FF) construct is measured through three dimensions such as Problem Solving (coded as DPM), consisting of items DPM1 to DPM8; Communication Skills (coded as DCS), consisting of items DCS9 to DCS16; and Personal Goals (coded as DPG), which reflect personal achievement within the family functioning context.

Psychological Vulnerability (PV) is measured using six items representing three core aspects such as need for validation, low self-esteem, and low independence. Meanwhile, (SMF) is assessed through three dimensions reflecting the impact of fatigue due to social media usage, namely, Cognitive Fatigue (coded as CG), Behavioral Fatigue (coded as B), and Emotional Fatigue (coded as Et), each consisting of five items. These dimensions capture mental burden, behavioral changes, and emotional responses arising from intensive social media interactions. The structure of this scale allows a comprehensive analysis of the relationship between, family functioning, and psychological vulnerability.

The measurement model in this study was designed as a reflective model, where indicators are seen as manifestations of latent constructs. In this model, the causal direction flows from the construct to the indicators. Therefore, any change in the construct is expected to be consistently reflected in all indicators that measure it. Additionally, indicators in a reflective model typically exhibit high intercorrelation, and removing one indicator does not significantly alter the meaning of the construct [44].

In this study, the Family Functioning (FF) construct is modeled reflectively through its three core dimensions, namely Problem Solving (DPM), Communication Skills (DCS), and Personal Goals (DPG). These dimensions reflect the extent of effective family functioning, aligning with the principle that indicators in a reflective model are effects, not formative causes, of the construct. Psychological Vulnerability (PV) is also modeled reflectively, where the six items used are considered to mirror three key aspects such as need for validation, low self-esteem, and dependence. Thus, these aspects are viewed as outcomes of an individual’s psychological vulnerability, rather than formative components [44]. Similarly, the (SMF) construct is developed as a second-order reflective construct comprising three dimensions with three dimensions consisting of Cognitive Fatigue (CG), Behavioral Fatigue (B), and Emotional Fatigue (Et). Each dimension is measured by five indicators reflecting users’ fatigue as a result of intensive social media use. This reflective-reflective structure is consistent with the multidimensional construct modeling approach recommended by [45], particularly in using the repeated indicators or disjoint two-stage approach for second-order modeling in PLS-SEM. Overall, the reflective approach enables the application of convergent validity tests (e.g., outer loading and AVE), internal reliability (composite reliability), and discriminant validity, as detailed in PLS-SEM guidelines [27].

The (SMF) and Family Functioning (FF) constructs are treated as second-order latent constructs. This approach is used because both constructs are multidimensional composed of several interrelated conceptual dimensions, each measured through specific indicators. For instance, SMF comprises three main dimensions: Cognitive Fatigue, Behavioral Fatigue, and Emotional Fatigue, each reflecting a different aspect of fatigue experienced from social media usage. Similarly, the FF construct includes the dimensions of Problem Solving, Communication Skills, and Personal Goals, which theoretically represent a comprehensive domain of family functioning.

According to [44]second-order reflective-reflective modeling can be used when a higher-order latent construct is formed by first-order reflective latent dimensions. This type of model, known as a hierarchical component model (HCM), is commonly used to more accurately capture complex conceptual structures in Partial Least Squares-based Structural Equation Modeling (PLS-SEM) [44]. also suggest that second-order models are particularly useful when researchers aim to analyze higher-order constructs directly without losing valuable information from their dimensional components.

#### Estimation process of second-order constructs

The constructs of (SMF) and Family Functioning (FF) were treated as second-order reflective constructs, each consisting of multiple first-order reflective dimensions. This approach allows researchers to manage the complexity of multidimensional constructs in a structured manner without sacrificing the conceptual depth of each dimension [44].

**Fig. 1:**
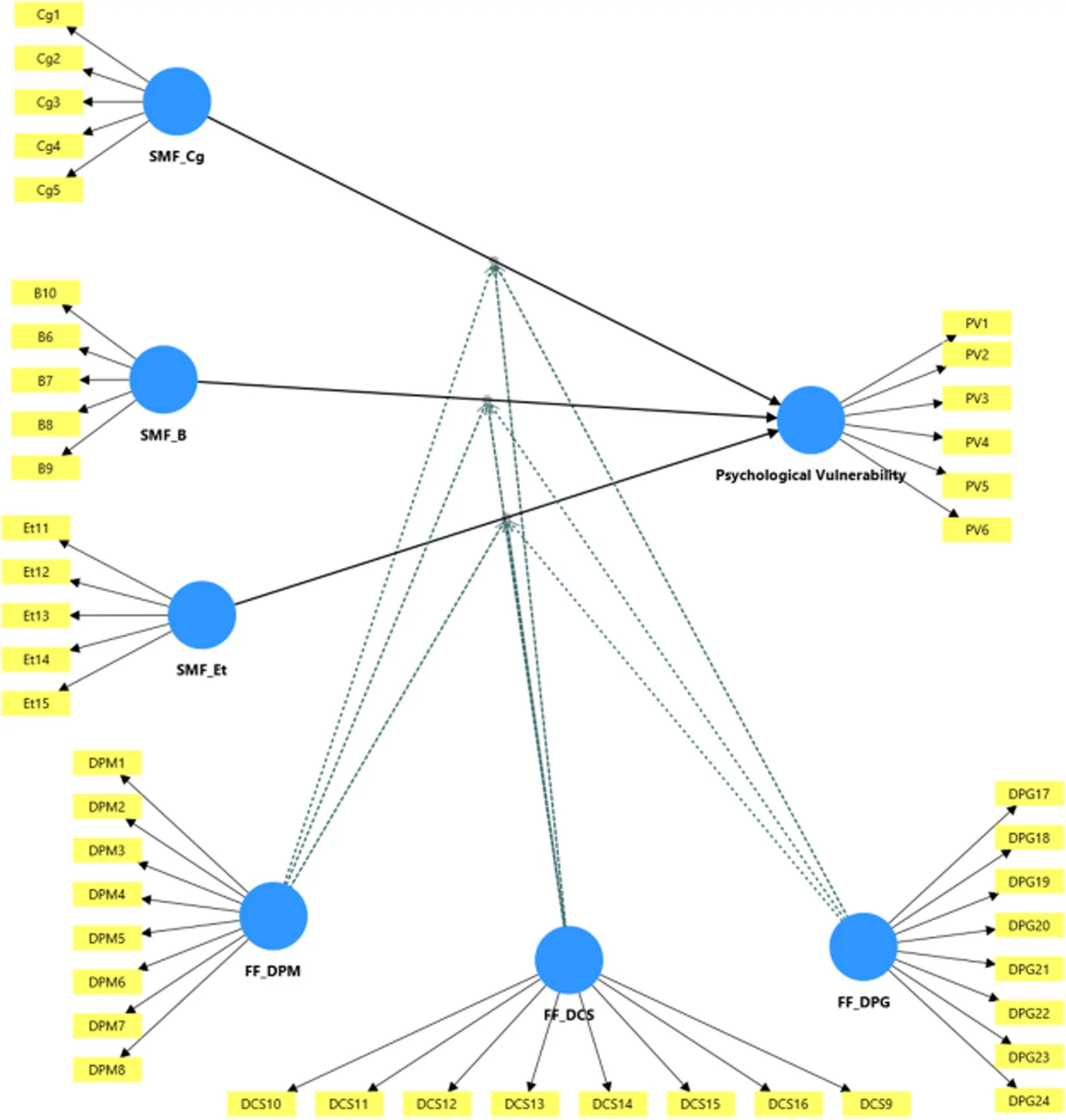
Estimation Process of Second-Order Constructs

To estimate these second-order constructs, this study employed the disjoint two-stage approach, as presented in Fig. 1. In the first stage, the measurement of first-order constructs was conducted, where each dimension of SMF (i.e., Cognitive Fatigue, Behavioral Fatigue, and Emotional Fatigue) and FF (i.e., Problem Solving, Communication Skills, and Personal Goals) was treated as a latent construct measured by its respective specific items. In this stage, the outer loadings of each item on its respective dimension were tested [44]. This stage also identified the latent variable scores for dimensions whose items had outer loadings equal to or greater than 0.70. These latent variable scores were then used as indicators in the second stage, serving as input to form the second-order constructs.

In the second stage, the SMF and FF constructs were treated as latent variables measured by the dimensions estimated in the previous stage. This approach is termed disjoint because the analysis of dimensions and second-order constructs occurs in two distinct and non-overlapping phases [44].The disjoint two-stage method was selected in this study due to its model simplicity and estimation efficiency. It is particularly recommended when the indicators of first- and second-order constructs are clearly structured and do not overlap [44]. This approach also facilitates the structural modeling of relationships between second-order latent constructs and other variables in the study. The process is illustrated in Fig. 1.

#### First-order construct analysis

### Stage 1 outer model

The measurement model was evaluated using the disjoint two-stage reflective–reflective approach as recommended by [46]. In Stage 1, the outer loadings of all first-order constructs were examined to ensure indicator reliability and convergent validity. Indicators with outer loadings below 0.70 were carefully evaluated. While loadings ≥ 0.70 are generally recommended, indicators with loadings between 0.40 and 0.70 may be retained if they are theoretically meaningful and do not adversely affect composite reliability or AVE. As shown in Table 5, all indicators of (SMF) and Family Functioning (FF) exceeded the 0.70 threshold, indicating satisfactory indicator reliability. For Psychological Vulnerability (PV), most items showed loadings between 0.65 and 0.77, while three items (PV1, PV2, PV6) had weaker loadings (< 0.50). Although the AVE value for Psychological Vulnerability (AVE = 0.374) is below the recommended threshold, the construct was retained due to its theoretical relevance and acceptable composite reliability (ρc = 0.773). This suggests that the construct maintains adequate internal consistency despite limited convergent validity. However, this limitation should be considered when interpreting the findings, and future research is recommended to refine the measurement of psychological vulnerability.

**Table 5:** Outer loadings of measurement items

Construct / Dimension	Item	Outer Loading	Interpretation
(SMF)			
Cognitive Fatigue (SMF_Cg)	Cg1	0.805	Reliable
	Cg2	0.904	Reliable
Behavioral Fatigue (SMF_B)	B7	0.799	Reliable
	B8	0.878	Reliable
	B9	0.904	Reliable
Emotional Fatigue (SMF_Et)	Et13	0.855	Reliable
	Et14	0.873	Reliable
	Et15	0.823	Reliable
Family Functioning (FF)			
Communication Skills (FF_DCS)	DCS12	0.708	Reliable
	DCS13	0.745	Reliable
	DCS14	0.704	Reliable
	DCS15	0.862	Reliable
	DCS16	0.858	Reliable
Personal Goals (FF_DPG)	DPG18	0.801	Reliable
	DPG19	0.736	Reliable
	DPG22	0.771	Reliable
	DPG23	0.829	Reliable
	DPG24	0.801	Reliable
Problem Solving (FF_DPM)	DPM1	0.819	Reliable
	DPM2	0.726	Reliable
	DPM3	0.847	Reliable
	DPM4	0.817	Reliable
	DPM5	0.819	Reliable
	DPM6	0.759	Reliable
	DPM7	0.735	Reliable
	DPM8	0.737	Reliable
Psychological Vulnerability (PV)			
	PV1	0.430	Below 0.50 (weak)
	PV2	0.475	Below 0.50 (weak)
	PV3	0.774	Acceptable
	PV4	0.657	Acceptable (below recommended threshold)
	PV5	0.747	Acceptable
	PV6	0.495	Marginal

Outer loadings ≥ 0.70 indicate acceptable indicator reliability; however, loadings between 0.40 and 0.70 may be retained based on theoretical justification and overall construct reliability [46].

While outer loadings ≥ 0.70 are generally recommended, indicators with loadings between 0.40 and 0.70 may be retained when their removal does not improve composite reliability or AVE and when they are theoretically meaningful (Hair et al., 2019; Hair et al., 2021). Therefore, the items were retained to preserve the conceptual integrity of the construct, as they reflect theoretically meaningful dimensions of psychological vulnerability that are essential for capturing the construct comprehensively. This decision is consistent with prior guidelines suggesting that indicators with loadings between 0.40 and 0.70 may be retained when their removal does not improve composite reliability or AVE and when they are theoretically meaningful [47–49].

### Construct reliability and validity

The construct reliability and validity were examined following the reflective measurement model evaluation guidelines by [46]. The evaluation covered internal consistency reliability measured through Cronbach’s alpha, rho_A, and composite reliability, as well as convergent validity assessed through the average variance extracted and discriminant validity evaluated using the Heterotrait–Monotrait ratio and the Fornell–Larcker criterion.

### Reliability and convergent validity

As summarized in Table 6, all constructs demonstrated acceptable levels of internal consistency reliability, with α, rho_A, and ρc exceeding 0.70. The AVE values for all constructs were above 0.50, indicating sufficient convergent validity, except for Psychological Vulnerability (AVE = 0.37). Although the AVE value for Psychological Vulnerability (AVE = 0.37) is below the recommended threshold, the construct was retained due to its theoretical relevance and acceptable composite reliability (ρc = 0.77). Previous methodological guidelines suggest that AVE values below 0.50 may still be acceptable when composite reliability is adequate and the construct is theoretically well-established. However, this limitation should be considered when interpreting the findings, and future research is encouraged to refine the measurement of psychological vulnerability.

**Table 6:** Construct reliability and convergent validity

Construct / Dimension	Cronbach’s α	rho_A	Composite Reliability (ρc)	AVE	Interpretation
Family Functioning (FF)					
Communication Skills (FF_DCS)	0.849	0.934	0.884	0.606	Reliable & valid
Personal Goals (FF_DPG)	0.855	0.925	0.891	0.621	Reliable & valid
Problem Solving (FF_DPM)	0.912	0.929	0.927	0.614	Reliable & valid
(SMF)					
Behavioral Fatigue (SMF_B)	0.827	0.847	0.896	0.743	Reliable & valid
Cognitive Fatigue (SMF_Cg)	0.643*	0.690	0.845	0.733	Reliable despite low α
Emotional Fatigue (SMF_Et)	0.812	0.832	0.887	0.724	Reliable & valid
Psychological Vulnerability (PV)	0.660	0.707	0.773	0.374	Reliable but AVE below 0.50

*α = 0.643

The reliability of the Cognitive Fatigue dimension (Cronbach’s α = 0.643) was slightly below the conventional threshold, indicating relatively greater measurement error than the other dimensions [50]. This may slightly reduce the precision of the higher-order Social Media Fatigue construct and its associated structural path estimates. However, the composite reliability of the higher-order construct remained satisfactory, supporting its retention for structural model analysis. Nevertheless, findings involving the Social Media Fatigue construct should be interpreted with appropriate caution, and future studies are encouraged to further refine the Cognitive Fatigue dimension to improve measurement precision.

### Discriminant validity

**Fig. 2:**
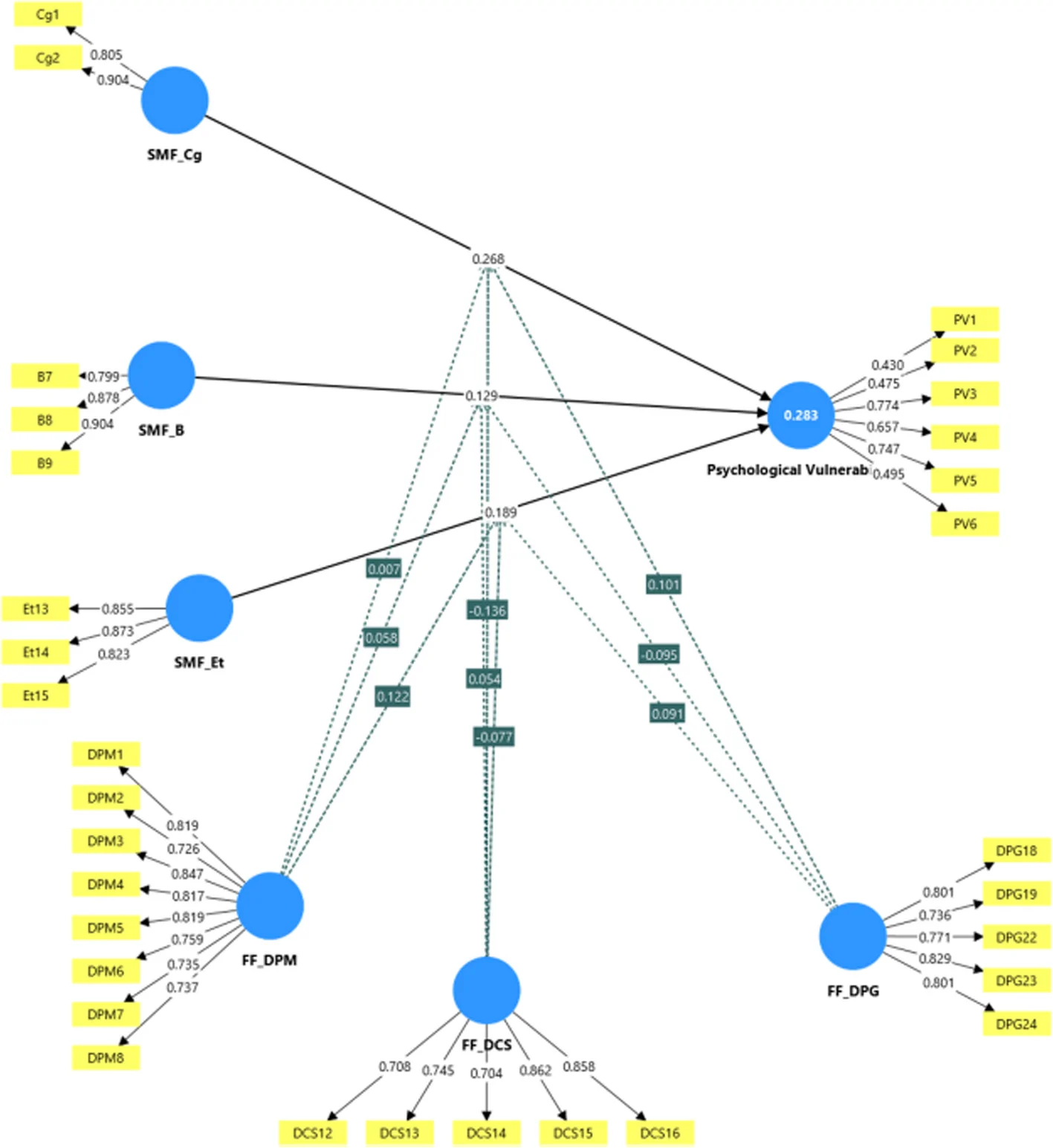
First-order construct analysis

Discriminant validity was tested using the HTMT ratio and Fornell–Larcker criterion. As shown in Table 7, all HTMT values were below 0.85, confirming discriminant validity among constructs [51]. Similarly, the Fornell–Larcker results indicated that the square roots of AVE (diagonal elements) were greater than their inter-construct correlations, verifying that each construct is empirically distinct Fig. 2.

**Table 7:** Discriminant validity results

Construct	FF_DCS	FF_DPG	FF_DPM	PV	SMF_B	SMF_Cg	SMF_Et
FF_DCS	0.779						
FF_DPG	0.740	0.788					
FF_DPM	0.744	0.730	0.784				
PV	–0.139	–0.170	–0.154	0.612			
SMF_B	–0.072	–0.073	–0.136	0.330	0.862		
SMF_Cg	–0.053	–0.081	–0.094	0.393	0.292	0.856	
SMF_Et	0.018	–0.027	–0.025	0.343	0.458	0.275	0.851

Diagonal elements (bold) represent the square roots of AVE values. All HTMT ratios < 0.85.

### Measurement model analysis for second-order construct

#### Measurement model analysis for second-order construct

The second stage of analysis followed the disjoint two-stage reflective–reflective approach recommended by Hair et al. (2021), integrating both first-order and higher-order constructs into a single PLS-SEM model. In this stage, *(SMF)* and *Family Functioning (FF)* were specified as second-order reflective constructs, each represented by the latent variable scores (LVS) of their respective first-order dimensions. Specifically, SMF was formed by the dimensions *Behavioral Fatigue (SMF_B)*, *Cognitive Fatigue (SMF_Cg)*, and *Emotional Fatigue (SMF_Et)*, whereas FF comprised *Communication Skills (FF_DCS)*, *Personal Goals (FF_DPG)*, and *Problem Solving (FF_DPM)*.

According to this approach, the latent variable scores from the lower-order constructs are used as manifest variables for estimating the higher-order constructs (Hair et al., 2021). Thus, each second-order construct is treated as a reflective latent variable, with its dimensions serving as indicators. Meanwhile, Psychological Vulnerability (PV) remained a first-order reflective construct, directly measured by three indicators (PV3, PV4, and PV5).

The evaluation of the second-order measurement model focused on the outer loadings between dimensions and their respective higher-order constructs. As shown in Table 8, all loadings were above the recommended threshold of 0.70, indicating acceptable convergent validity and stable measurement properties.

**Table 8:** Outer Loadings of Second-Order Constructs

Construct	Dimension / Indicator	Outer Loading
Family Functioning (FF)	FF_DCS	0.905
	FF_DPG	0.917
	FF_DPM	0.902
Psychological Vulnerability (PV)	PV3	0.852
	PV4	0.690
	PV5	0.772
(SMF)	SMF_B	0.743
	SMF_Cg	0.742
	SMF_Et	0.758

All outer loadings are statistically significant (*p* < .001)

In *Psychological Vulnerability*, indicators PV3 and PV5 showed acceptable and statistically significant loadings (0.852 and 0.772; *p* < .001). PV4 demonstrated a slightly lower loading (0.690; *p* < .001) but was retained because it theoretically represents a critical facet of self-evaluation within the vulnerability construct. The construct’s reliability (ρc = 0.77; α = 0.66) met the minimum acceptable standards, supporting its inclusion in the model. For the second-order constructs, all dimensions of SMF and FF demonstrated statistically significant loadings ranging from 0.74 to 0.92, confirming that each dimension reliably reflects its higher-order construct.

### Construct reliability and validity

The reliability and validity of the second-order constructs were assessed using Composite Reliability (CR), Cronbach’s Alpha, Rho_A, and Average Variance Extracted (AVE). As shown in Table 9, all constructs exceeded the recommended threshold for composite reliability (ρc ≥ 0.70) and AVE (≥ 0.50), indicating satisfactory internal consistency and convergent validity.

**Table 9:** Construct Reliability and Validity

Construct	Cronbach’s Alpha	rho_A	Composite Reliability (ρc)	AVE
Family Functioning	0.894	0.903	0.934	0.825
Psychological Vulnerability	0.669	0.707	0.817	0.599
	0.609	0.608	0.792	0.559

Although the Cronbach’s Alpha and Rho_A values for SMF were slightly below 0.70, the overall reliability remained acceptable for exploratory and predictive modeling contexts [44]. The *Family Functioning* construct demonstrated relatively strong psychometric performance (α = 0.894; ρc = 0.934; AVE = 0.825), whereas *Psychological Vulnerability* showed moderate reliability but adequate validity (ρc = 0.817; AVE = 0.599).

The evaluation results confirm that the second-order measurement model meets the essential reliability and validity criteria. Among the constructs, *Family Functioning* demonstrated the highest reliability and convergent validity, indicating that this construct was measured with precision and stability. *Psychological Vulnerability* achieved the minimum acceptable reliability thresholds and exhibited conceptual completeness, supporting its adequacy as a first-order construct within the model. Meanwhile, showed acceptable convergent validity, although its reliability values were moderately lower. This finding may reflect the inherently multidimensional nature of the construct, which can lead to slightly reduced internal consistency. Furthermore, discriminant validity assessment using the Heterotrait–Monotrait ratio (HTMT) showed that all inter-construct values were below 0.85 (e.g., FF–PV = 0.253; SMF–PV = 0.676), indicating that each construct is empirically distinct and free from measurement overlap. Overall, these results confirm that both first- and second-order constructs possess adequate psychometric properties and are valid for subsequent structural model analysis Figs. and 4. 

**Fig. 3:**
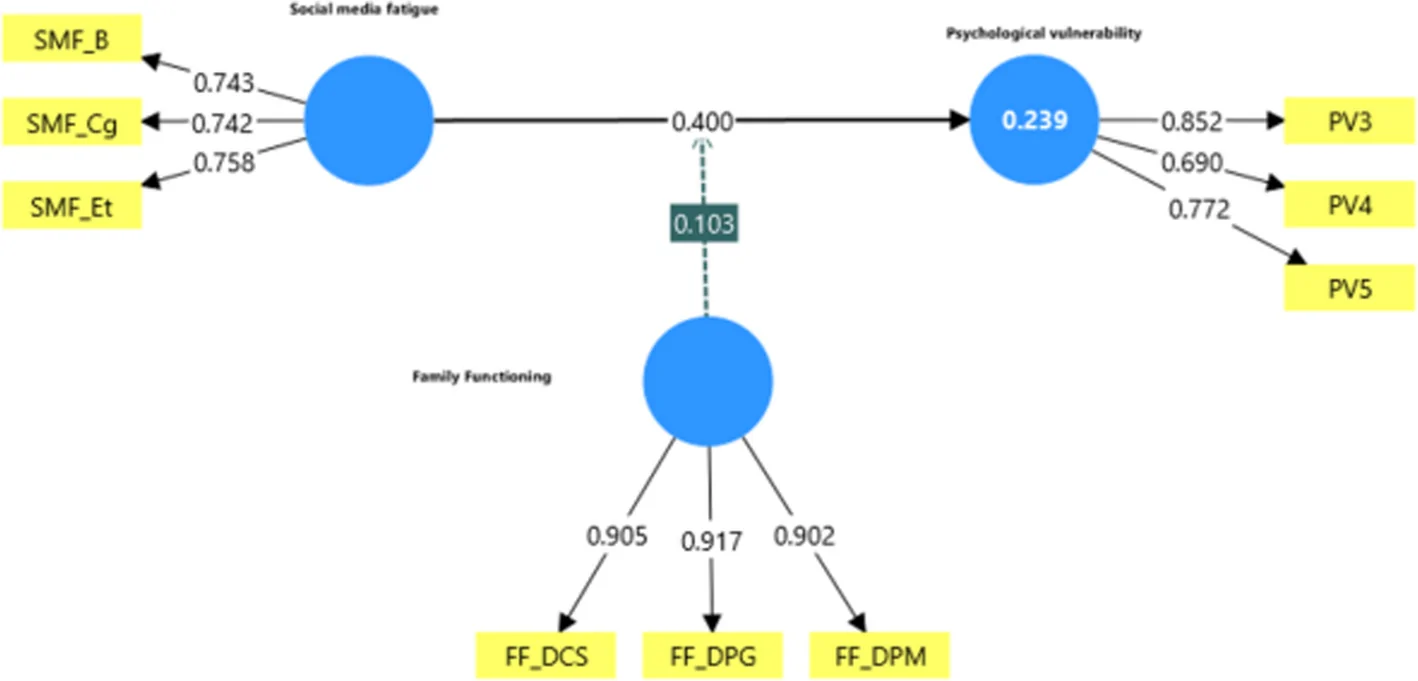
Outer Model Evaluation

#### Evaluasi inner model (model struktural)

## Results

### Descriptive statistics and correlation analysis

Prior to testing the structural model, descriptive and correlational analyses were conducted to examine the distribution and relationships among the key study variablesconsisting of (SMF), Psychological Vulnerability (PV), and Family Functioning (FF). The results in Table 3 show that all variables demonstrated approximately normal distributions, with skewness and kurtosis values within the acceptable range (± 1). The results in Table 3 show that all variables demonstrated approximately symmetric distributions, with skewness and kurtosis values within the acceptable range (± 1). These statistics are reported for descriptive purposes. Because PLS-SEM is robust to non-normal data, strict multivariate normality is not required for the estimation procedure.

Pearson’s correlation analysis (Table 3) revealed a significant positive association between and Psychological Vulnerability (*r* = .382, *p* < .001), indicating that higher levels of digital fatigue were associated with greater psychological vulnerability. Family Functioning showed weak, negative correlations with both variables, suggesting that supportive family dynamics may slightly reduce the adverse psychological effects of social media use.

### Structural model evaluation

**Fig. 4:**
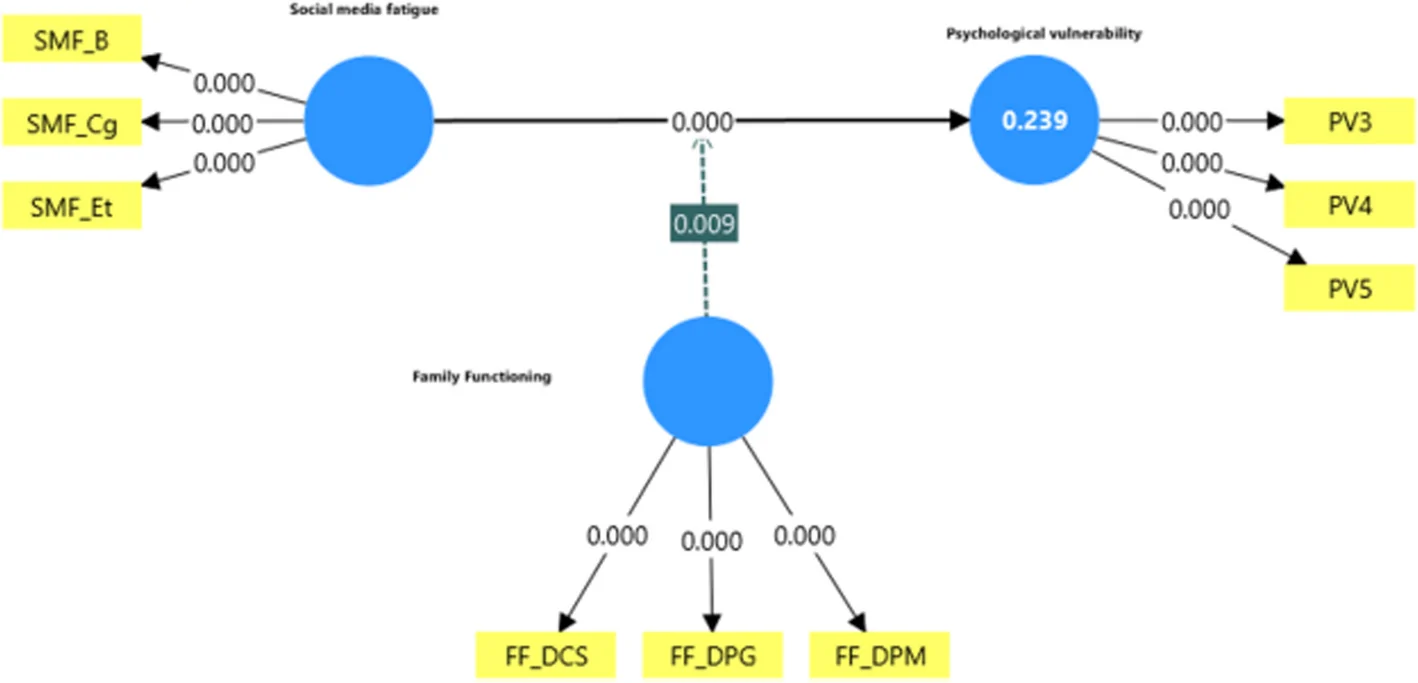
Inner model (hyphothesis testing)

The inner structural model is illustrated in Fig. 4. The results reveal a significant negative path from Family Functioning to Psychological Vulnerability (β = − 0.174, t = 3.426, *p* = .001), indicating that higher family functioning is associated with lower psychological vulnerability. Conversely, demonstrates a significant positive relationship with Psychological Vulnerability (β = 0.400, t = 8.718, *p* < .001), confirming that greater digital fatigue predicts higher vulnerability.

Furthermore, the interaction between Family Functioning and was significant (β = 0.103, *t* = 2.621, *p* = .009; see Table 10E), suggesting a moderating effect. Thus, family functioning modifies the strength of the association between and psychological vulnerability.

**Table 10:** Path coefficients and hypothesis testing results

Hypothesis	β (Original Sample)	SD	t	*p*
Family Functioning → Psychological Vulnerability	–0.174	0.051	3.426	0.001
Family Functioning × → Psychological Vulnerability	0.103	0.039	2.621	0.009
→ Psychological Vulnerability	0.400	0.046	8.718	< 0.001

### Conditional direct effects

A conditional direct effect analysis was conducted to further examine how Family Functioning moderates the relationship between and Psychological Vulnerability across three levels of the moderator variable. The results revealed that when Family Functioning was high (+ 1 SD), the effect of on Psychological Vulnerability was relatively stronger (β = 0.503, t = 10.730, *p* < .001). When Family Functioning was at an average level, the effect remained significant (β = 0.400, t = 8.718, *p* < .001), whereas when Family Functioning was low (–1 SD), the effect was comparatively weaker though still statistically significant (β = 0.298, t = 4.170, *p* < .001).

These results indicate that family functioning shows a protective main effect through its direct negative association with psychological vulnerability. However, its moderating role does not operate in a buffering direction. Instead, the relationship between and psychological vulnerability becomes stronger at higher levels of family functioning, suggesting a context-dependent and non-linear interaction pattern. This finding highlights the complexity of family processes in the digital context and indicates that the moderating influence of family functioning cannot be assumed to be uniformly protective.

### Model fit and effect size

The model demonstrated acceptable fit and explanatory power, as shown in Table 11. The coefficient of determination for Psychological Vulnerability was R² = 0.239, with an adjusted value of 0.231, indicating that approximately 23.9% of the variance in Psychological Vulnerability was explained by the predictors and Family Functioning. This represents a moderate level of explanatory power according to Chin’s (1998) classification. Model fit indices further supported the adequacy of the structural model. The Standardized Root Mean Square Residual (SRMR) values for both the saturated and estimated models were 0.095, falling below the threshold of 0.10 but approaching the more conservative cutoff of 0.08, indicating a borderline fit. The Normed Fit Index (NFI) was 0.736, reflecting a modest level of fit. The discrepancy measures (d < sub> ULS</sub > = 0.406; d < sub> G</sub > = 0.130) indicated relatively small differences between the observed and model-implied covariance matrices.

**Table 11:** Effect size and model fit indices

Criterion	Value
R² (Psychological Vulnerability)	0.239
Adjusted R²	0.231
SRMR (Saturated / Estimated)	0.095 / 0.095
NFI	0.736
d_ULS / d_G	0.406 / 0.130

Taken together, these results suggest that the proposed model provides a reasonably good representation of the data, with acceptable fit indices and moderate explanatory power for the dependent variable, Psychological Vulnerability. Further examination of effect sizes (f²) revealed that the contribution of each predictor varied in strength as seen in Table 12.

**Table 12:** Effect Size (f²) Matrix

Predictor Variable	Family Functioning	Psychological Vulnerability	Family Functioning ×
Family Functioning		0.039	
Psychological Vulnerability			
		0.200	
Family Functioning ×		0.016	

*f²* values indicate the local effect size of each predictor on the dependent variable (*Psychological Vulnerability*)

The effect of Family Functioning on Psychological Vulnerability yielded an f² value of 0.039, which falls within the small category. Despite its modest magnitude, this effect remains theoretically meaningful because it reflects the role of family functioning as a protective factor that helps reduce vulnerability to digital stressors., on the other hand, demonstrated an f² value of 0.200, which is categorized as medium, indicating that fatigue from continuous digital exposure exerts a substantial impact on individuals’ psychological vulnerability and is the most dominant predictor in the model. Meanwhile, the interaction term representing the moderation between Family Functioning and produced an f² value of 0.016 classified as very small but statistically significant. This result shows that while the moderating effect of Family Functioning is limited in strength, it still contributes uniquely to explaining the variance in psychological vulnerability.

## Discussion

The results confirm that significantly predicts Psychological Vulnerability, consistent with prior evidence that digital overload heightens stress, anxiety, and emotional instability. Continuous exposure to online content, social comparison, and information saturation imposes cognitive and emotional strain, thereby weakening psychological resilience [52–54]. The present findings are also consistent with recent evidence suggesting that the psychological consequences of social media use depend not only on the amount of use but also on how individuals engage with online platforms [16]. Active social media use, characterized by direct interaction and communication, has generally been associated with greater social connectedness and more favorable psychological outcomes, whereas passive consumption of social media content has been linked to increased social comparison, emotional exhaustion, and poorer well-being [16]. More recent evidence further suggests that passive forms of social media engagement are associated with greater emotional strain and less favorable mental health outcomes [17].

Conversely, *Family Functioning* demonstrated a significant negative association with *Psychological Vulnerability*, supporting the notion that healthy family systems buffer stress through emotional support, open communication, and adaptive coping [55]. Such environments foster self-regulation, validation, and a sense of security that mitigate the adverse effects of digital overload [56].

However, the moderation analysis revealed a context-dependent pattern in which the association between and psychological vulnerability was strongest at higher levels of family functioning. Although statistically significant, the moderation effect was very small (f² = 0.016), indicating limited practical significance and should be interpreted with caution. Although this study is among the first to examine the moderating role of family functioning in the relationship between and psychological vulnerability, the findings can be understood within the broader literature on stress and protective factors. Previous studies have consistently shown that family-related factors and social support may serve as buffers against psychological distress, although the strength of these effects varies across contexts [57, 58].

This suggests that the moderating role of family functioning does not follow a simple buffering mechanism. While family functioning demonstrated a protective direct effect, its interaction with may reflected a non-buffering pattern.

From a family systems perspective, this pattern may reflect the dynamics of emotional interdependence within highly cohesive families [59, 60]. In such systems, emotional states are often shared across members, and stress experienced by one individual may resonate throughout the family unit [60, 61]. Under conditions of digital fatigue, this shared emotional climate may intensify sensitivity to stressors rather than attenuate them [62]. Highly cohesive families may also involve increased monitoring, shared digital concerns, or collective reactions to online experiences, which can heighten emotional arousal instead of reducing it [63, 64]. Thus, the same characteristics that typically support well-being may, in certain digital contexts, contribute to greater stress reactivity [65, 66].

It is important to distinguish between the two roles of family functioning observed in this study. As a direct predictor, higher family functioning was associated with lower psychological vulnerability, indicating a protective main effect. In contrast, its moderating role was non-buffering, as the relationship between and vulnerability became stronger at higher levels of family functioning. This conceptual distinction may help prevent misinterpretation of family functioning as uniformly protective across all analytical contexts [65]. Another consideration when interpreting the moderation findings relates to the demographic composition of the sample, which consisted predominantly of female university students. Previous research suggests that young women tend to engage with social media differently from men, often reporting higher levels of emotional involvement, social comparison, and sensitivity to online interpersonal feedback. Studies have also indicated that females may experience stronger psychological consequences associated with social media engagement, including greater susceptibility to depressive symptoms and digital stress. Therefore, the relationships observed in the present study may partly reflect characteristics that are particularly relevant to young female students. This demographic composition may have contributed to the observed pattern of associations and should be considered when interpreting the present findings.

An alternative explanation is that the observed moderation pattern may partly reflect measurement limitations. The Family Functioning Questionnaire primarily assesses competencies such as communication, problem solving, and goal orientation, but may not fully differentiate healthy cohesion from excessive interdependence or enmeshment [67]. Consequently, higher scores could reflect strong involvement alongside heightened emotional interdependence, which may amplify stress responses in certain digital contexts [50]. Therefore, the very small moderation effect observed in the present study may reflect a combination of the relatively homogeneous demographic composition of the sample, which consisted predominantly of female university students, and the limited ability of the FFQ to capture family boundary processes such as enmeshment that may be more directly relevant to buffering the psychological consequences of. Future research should incorporate instruments that assess family boundaries and enmeshment more explicitly, use multi-informant designs, and apply longitudinal approaches to clarify whether the observed pattern reflects a theoretical family-systems process or a measurement artefact.

## Theoretical and practical implications

### Theoretical implications

This study contributes to the literature by integrating and family functioning into a single structural model of psychological vulnerability. The findings support the role of digital fatigue as a psychological stressor while also highlighting the complex, context-dependent role of family systems. Rather than functioning solely as a buffering factor, family functioning appears to operate through both a protective direct effect and a non-buffering moderating mechanism. This distinction expands the vulnerability–stress framework by suggesting that family processes in the digital era may shape psychological outcomes in nonlinear ways.

### Practical implications

Given the moderate explanatory power of the model (R² = 0.239) and the very small magnitude of the interaction effect (f² = 0.016), the practical implications of these findings should be interpreted cautiously. Rather than implying a strong intervention effect, the results primarily support preventive and awareness-oriented strategies.

For families, the findings highlight the importance of open communication about online experiences, as well as the need to balance emotional support with respect for individual autonomy and digital boundaries. For educators and policymakers, promoting digital literacy and awareness of may help individuals recognize early signs of psychological strain. For mental-health practitioners, may serve as a useful indicator of digital stress that warrants screening and psychoeducational support, rather than as a standalone target for intensive intervention.

## Limitations and future directions

Several limitations should be acknowledged. First, the cross-sectional design restricts causal inference. Longitudinal studies are needed to examine how, family functioning, and psychological vulnerability evolve over time.

Second, the study relied exclusively on self-report measures collected through an online survey, which may introduce response bias and common method variance. Future studies may benefit from incorporating multi-informant assessments, behavioral indicators, or mixed-methods approaches to obtain a more comprehensive understanding of digital experiences and family dynamics.

Third, the use of convenience sampling and the demographic composition of the sample limit the generalizability of the findings. Participants were recruited through online distribution channels and consisted predominantly of young adult university students, with females accounting for 77.1% of the sample. Previous research suggests that young women tend to engage with social media differently from men, often reporting higher levels of emotional involvement, social comparison, and sensitivity to online interpersonal feedback [56]. These differences may contribute to greater susceptibility to certain forms of digital stress and psychological strain [57]. Consequently, the present findings should be interpreted as primarily reflecting the experiences of a predominantly female young-adult sample from Pekanbaru rather than the broader adult population. Future studies should recruit more diverse and balanced samples across age groups, educational backgrounds, and gender categories and examine potential demographic differences through multi-group analyses.

Fourth, the measurement of Psychological Vulnerability demonstrated relatively modest convergent validity, which may affect the precision of the construct and should therefore be interpreted with caution. Finally, although the moderating effect of family functioning was statistically significant, its effect size was very small (f² = 0.016), indicating limited practical significance. Accordingly, the moderating role of family functioning should be interpreted cautiously and warrants further investigation in future studies.

## Conclusion

This study examined the structural relationship between, family functioning, and psychological vulnerability. The results indicate that is a significant risk factor for psychological vulnerability, while family functioning shows a protective direct association with lower vulnerability. However, the moderating effect of family functioning was small and non-buffering, with the association between and vulnerability becoming stronger at higher levels of family functioning.

These findings suggest that the role of family systems in digital-era mental health may be more complex than traditional buffering models imply. Given the cross-sectional design, moderate explanatory power, and potential measurement limitations, the results should be interpreted as preliminary and context-specific. Future research using longitudinal designs, boundary-sensitive family measures, and multi-informant data is needed to clarify the mechanisms underlying these relationships.

## Supplementary Information

Supplementary Material 1.

Supplementary Material 2.

Supplementary Material 3.

Supplementary Material 4.

Supplementary Material 5.

## Data Availability

The datasets generated and/or analyzed during the current study are not publicly available due to participant confidentiality and ethical restrictions imposed by the Ethics Committee of the Faculty of Psychology, Universitas Islam Riau (Approval No: 023/EC-FPUIR/IV/2025). De-identified data may be available from the corresponding author (L.N.) on reasonable request for research purposes and in compliance with ethical guidelines.
