# Supplementary material for: A structural model of psychological vulnerability in the era of social media
Source: Discov Ment Health. 2026 Jul 6;6(1):181. doi: 10.1007/s44192-026-00541-1 (PMC13620041; doi:10.1007/s44192-026-00541-1)
Supplement: Supplementary file 1 [file 44192_2026_541_MOESM1_ESM.pdf]

UNIVERSITAS ISLAM RIAU
FAKULTAS PSIKOLOGI
الجامعة الإسلامية الريوية

Alamat : Jalan Kaharuddin Nasution No. 113, Marpoyan, Pekanbaru, Riau, Indonesia - 28284
Telp. +62 761 674674 Fax. +62 761 674834 Email : psikologi@uir.ac.id Website : www.uir.ac.id

Ethics Approval Statement

No. : 023/EC-FPUIR/IV/2025
Date : 4 February 2025
Title of Study :A Structural Model of Psychological Vulnerability in the Era of Social Media

Principal Investigator:
Lisfarika Napitupulu
Ethics Committee:
Ethics Committee of the Faculty of Psychology, Universitas Islam Riau

This study received ethical approval from the Ethics Committee of the Faculty of Psychology, Universitas Islam Riau. All research procedures involving human participants were conducted in accordance with the ethical standards and regulations of the committee and with the principles stated in the Declaration of Helsinki. Prior to participation, all respondents were informed about the objectives, procedures, potential risks, and benefits of the study. Written informed consent was obtained from each participant. Confidentiality and anonymity were strictly maintained throughout the study, and participants retained the right to withdraw from the study at any time without penalty.

On behalf of the Ethics Committee
Faculty of Psychology, Universitas Islam Riau

UNIVERSITAS ISLAM RIAU
FAK. PSIKOLOGI

Dr. Fikri, M.Si
Chairperson, Ethics Committee
Faculty of Psychology, Universitas Islam Riau
