# Supplementary material for: A structural model of psychological vulnerability in the era of social media
Source: Discov Ment Health. 2026 Jul 6;6(1):181. doi: 10.1007/s44192-026-00541-1 (PMC13620041; doi:10.1007/s44192-026-00541-1)
Supplement: Supplementary file 2 [file 44192_2026_541_MOESM2_ESM.docx]

**Declarations**

*Ethical approval*

This study received ethical approval from the Ethics Committee of the Faculty of Psychology, Universitas Islam Riau (Approval No: 023/EC-FPUIR/IV/2025, dated 4 February 2025). All procedures were conducted in accordance with the Declaration of Helsinki (2013) and the American Psychological Association Ethical Principles of Psychologists and Code of Conduct (2017).

*Consent to participate*

All participants were fully informed about the study’s objectives, procedures, confidentiality, potential risks, and benefits. Written informed consent was obtained prior to participation. Participation was voluntary, and participants retained the right to withdraw at any time without penalty. Confidentiality and anonymity were strictly maintained throughout the study.

*Consent to publish*

Not applicable. No identifying personal data, images, or videos are included in this manuscript that would require explicit consent for publication.
