# Supplementary material for: A structural model of psychological vulnerability in the era of social media
Source: Discov Ment Health. 2026 Jul 6;6(1):181. doi: 10.1007/s44192-026-00541-1 (PMC13620041; doi:10.1007/s44192-026-00541-1)
Supplement: Supplementary file 3 [file 44192_2026_541_MOESM3_ESM.docx]

**Abstract**

Population description is vague; the age range and student-dominated sample should be acknowledged.

“Context-dependent moderation pattern” is imprecise and should be clarified statistically.

The interaction effect (f² = .016) should be explicitly described as very small.

Minor repetition and keyword punctuation need correction.

**Introduction**

Reduce redundancy and duplicate sentences on psychological vulnerability.

The research gap is not sharply articulated; clarify what is uniquely unknown.

Justify the choice of family functioning over other protective factors.

H3 is non-directional and requires a stronger theoretical specification.

Minor language and citation formatting issues present.

**Methods**

The authors claim the use of random sampling, yet participants were recruited through online group distribution. Please clarify the exact sampling procedure and explain how randomness was ensured. Otherwise, the sampling method should be more accurately described as non-probability sampling

Major inconsistency: claimed random sampling vs. convenience-based outreach.

The sample is highly skewed (mostly young students, predominantly female); external validity is limited and should be explicitly discussed.

Common method bias was not assessed.

Clarify the online consent procedure.

Provide clearer cultural/linguistic validation of PVS and FFQ.

**Measurement Model**

Psychological Vulnerability shows weak loadings and low AVE; a stronger justification or explicit limitation is needed.

Internal inconsistency regarding outer loading thresholds should be corrected.

Tone in some sections is overly defensive.

Cognitive Fatigue reliability is borderline and should be noted.

**Structural Results**

Important control variables were not included in the model.

The moderation effect is statistically significant but practically very small; avoid over-interpretation.

Model fit claims should be more cautious (SRMR borderline; NFI modest).

**Discussion & Limitations**

Some proposed mechanisms are speculative and require stronger hedging.

Better integration with prior moderation literature is needed.

Practical implications are somewhat generic.

Limitations should explicitly note convenience sampling, student bias, weak PV measurement, and a very small interaction effect.

**Formatting**

Correct duplicate Results numbering.

Standardize spacing and statistical notation.
